# Multicenter multiplatform pattern-of-practice analysis of single-isocenter multitarget stereotactic radiosurgery

**DOI:** 10.1007/s00066-025-02424-w

**Published:** 2025-07-10

**Authors:** Benedikt Thomann, Tobias Fechter, Johannes Fischer, Armin Runz, Julian Roers, Ute Ludwig, Melanie Grehn, Maximilian Grohmann, Christian Ziemann, Michael Judge, Wolfgang Baus, Michelle Grahle, Matthias Walke, Bastian Bathen, Janett Köhn, Paul Käthner, Maya Shariff, Rebecca Matthis, Jens Fleckenstein, Sascha Großmann, Tino Streller, Simon Howitz, Marlen Priegnitz, Rocco Weigel, Peter Winkler, Oliver Blanck, Daniela Schmitt, Jurgen Beck, Marcia Machein, Evangelos Pappas, Ilinca Popp, Michael Reiner, Christian P. Karger, Christos Moustakis, Michael Bock, Anca-Ligia Grosu, Dimos Baltas

**Affiliations:** 1 Klinik für Strahlenheilkunde, Abteilung Medizinische Physik, Universitätsklinikum Freiburg, Freiburg, Germany; 2 Medizinische Fakultät, Albert-Ludwigs-Universität Freiburg, Freiburg, Germany; 3 Partnerstandort Freiburg, Deutsches Konsortium für Translationale Krebsforschung (DKTK), Freiburg, Germany; 4 Partnerstandort Freiburg, Deutsches Krebsforschungszentrum (DKFZ), Freiburg, Germany; 5 Klinik für Radiologie, Medizinische Physik, Universitätsklinikum Freiburg, Freiburg, Germany; 6 Abteilung Medizinische Physik in der Strahlentherapie, Deutsches Krebsforschungszentrum (DKFZ), Heidelberg, Germany; 7 Heidelberger Institut für Radioonkologie (HIRO), National Center for Radiation Research in Oncology (NCRO), Heidelberg, Germany; 8 Klinik für Strahlentherapie und Radioonkologie, Universitätsklinikum Münster, Münster, Germany; 9 Klinik für Strahlentherapie, Universitätsklinikum Schleswig-Holstein, Kiel, Germany; 10 Saphir Radiochirurgie Zentrum Frankfurt am Main und Norddeutschland, Kiel, Germany; 11 Klinik für Strahlentherapie und Radioonkologie, Universitätsklinikum Hamburg-Eppendorf, Hamburg, Germany; 12 Klinik für Strahlentherapie, Universitätsklinikum Schleswig-Holstein, Lübeck, Germany; 13 Klinik und Poliklinik für Radioonkologie, Cyberknife- und Strahlentherapie, Uniklinik Köln, Köln, Germany; 14 Strahlentherapie, Wege Klinik Bonn, Bonn, Germany; 15 Strahlentherapie, Medizinische Physik, Städtisches Klinikum Dresden, Dresden, Germany; 16 Medizinische Physik, Universitätsklinik für Strahlentherapie Magdeburg, Magdeburg, Germany; 17 Klinik für Strahlentherapie und Onkologie, Medizinische Physik, Universitätsmedizin Frankfurt, Frankfurt, Germany; 18 Klinik und Poliklinik für Strahlentherapie und Radioonkologie, Uniklinikum Würzburg, Würzburg, Germany; 19 Klinik für Radioonkologie, Universitätsklinikum Erlangen, Friedrich-Alexander-Universität Erlangen-Nürnberg (FAU), Erlangen, Germany; 20 Comprehensive Cancer Center Erlangen-EMN (CCC ER-EMN), Erlangen, Germany; 21 Klinik für Strahlentherapie und Radioonkologie, Medizinische Physik, Klinikum Stuttgart, Stuttgart, Germany; 22 Klinik für Strahlentherapie und Radioonkologie, Universitätsmedizin Mannheim, Mannheim, Germany; 23 Klinik und Poliklinik für Radioonkologie und Strahlentherapie, Universitätsmedizin Mainz, Mainz, Germany; 24 Radioonkologie, Medizinphysik, Luzerner Kantonsspital, Luzern, Switzerland; 25 Strahlentherapie und Radioonkologie, SRH Wald-Klinikum Gera, Gera, Germany; 26 Klinik für Radioonkologie und Strahlentherapie, Medizinische Universität Lausitz—Carl Thiem, Cottbus, Germany; 27 Universitätsklinik für Strahlentherapie und Radioonkologie, Tirol Kliniken, Innsbruck, Austria; 28 Abteilung für Therapeutische Radiologie und Onkologie, Medizinische Universität Graz, Graz, Austria; 29 Klinik für Strahlentherapie und Radioonkologie, Universitätsmedizin Göttingen, Göttingen, Germany; 30 Comprehensive Cancer Center, Universitätsmedizin Göttingen, Göttingen, Germany; 31 Klinik für Neurochirurgie, Universitätsklinikum Freiburg, Freiburg, Germany; 32 Department of Biomedical Sciences, Radiology and Radiotherapy Sector, University of West Attica, Athens, Greece; 33 Klinik für Strahlenheilkunde, Universitätsklinikum Freiburg, Freiburg, Germany; 34 Strahlentherapie und Radioonkologie, Medizinische Physik, LMU Klinikum München, München, Germany; 35 Klinik und Poliklinik für Strahlentherapie, Medizinische Physik, Universitätsklinikum Leipzig, Leipzig, Germany

**Keywords:** SIMT SRS, Brain metastases, Anthropomorphic phantom, Ring trial, End-to-end test

## Abstract

**Purpose:**

Single-isocenter multitarget stereotactic radiosurgery (SIMT SRS) offers enhanced clinical efficiency for treating multiple brain metastases. However, it introduces additional uncertainties, such as off-center dose and beam profile inaccuracies, as well as quality assurance (QA) challenges, complicating its implementation. This study aims to evaluate different SIMT SRS approaches.

**Methods:**

We collected and analyzed SIMT SRS protocol and infrastructure parameters from 23 radiotherapy centers across Germany, Austria, and Switzerland, encompassing immobilization systems, computed tomography (CT) protocols, linear accelerators, treatment planning systems, beam configurations, imaging techniques, and QA practices. Consensus, deviations, and compliance with current guidelines were assessed. Subsequent studies will include on-site measurements, evaluation of treatment plan quality and delivery accuracy, and correlation of these findings with the analyzed protocols to identify potential links between protocol parameters and clinical outcomes.

**Results:**

There is consensus (at least 80% agreement) for a CT slice thickness of ≤ 1 mm, the need for six-degree-of-freedom patient setup correction, and noncoplanar treatment. There is notable variability for intrafraction imaging (used by 70%), minimum accepted planning target volume diameter (ranging from 2–10 mm), SRS QA, and general plan parameters, such as photon energy and number of treatment fields. There is also high variability in employed linear accelerator models and treatment planning systems.

**Conclusion:**

These findings highlight a lack of standardization in SIMT SRS practices. Combined with future measurements correlating protocols to treatment quality, our study will provide a foundation for recommendations to support the safe and standardized implementation of SIMT SRS.

## Introduction

According to latest guidelines, stereotactic radiosurgery (SRS) is a recommended treatment approach for patients with 1–3 but also 4–10 brain metastases [1, 2]. Several radiotherapy (RT) systems dedicated or modified for SRS are in clinical use [3, 4]. However, the number of SRS treatments and the number of brain metastases per treatment is continuously increasing due to novel drug developments and rising clinical evidence of reduced toxicity compared to whole brain RT [5–8]. Practically, every RT center may face the necessity to offer multitarget SRS for their patients soon, mainly with standard C‑arm RT systems specially modified for SRS treatment. Technically, multiple lesions can be treated consecutively, targeting each lesion separately with individual treatment planning, patient setup and delivery procedures. This single-target SRS is already used by many radiotherapy centers because of its comparatively simple implementation, well-known and easily assessable uncertainties and well-published clinical outcome [9, 10].

With increasing number of brain metastases, however, single-target SRS becomes less efficient and feasible. This led to the development of single-isocenter multitarget (SIMT) SRS, in which multiple lesions are targeted simultaneously with a single treatment plan and isocenter [11, 12]. SIMT SRS enhances treatment planning and efficiency and allows for the treatment of all metastases instead of a selected subset while preserving the benefits of SRS. However, it also introduces potential additional uncertainties for two main reasons: (1) standard quality assurance (QA) practices are primarily focused on the isocenter and sometimes neglect off-centered fields; (2) off-center uncertainties, influenced by collimator and couch rotations, may increase with distance from the isocenter, making them harder to predict and quantify. Determining the overall uncertainty in SIMT SRS is complex and may depend on the specific techniques, systems, and protocols used at the radiotherapy center.

Considering these challenges and uncertainties, it can be difficult for RT centers to decide whether to implement SIMT SRS and, equally important, which techniques and protocols to use. At the same time, there is a large variety of techniques and protocols already in place across RT centers that have started using SIMT SRS.

To our knowledge, there are no specific regulations or guidelines for SIMT SRS, and existing stereotactic guidelines allow for some flexibility in this regard [3, 4] (e.g., while end-to-end testing is recommended, the specifics of its implementation remain unclear). As a project of the working group for stereotactic radiotherapy of the German Society for Medical Physics (WG STX DGMP) in close collaboration with the working groups for neuroradio-oncology and for stereotactic radiotherapy of the German Society for Radiation Oncology (DEGRO), our German Cancer Aid funded study aims to illustrate how existing stereotactic guidelines are applied for SIMT SRS and to assess the degree of flexibility they leave to individual centers.

For this purpose, we investigate the infrastructure and protocols across 23 RT centers in Germany, Switzerland, and Austria, focusing on variations in setup and imaging techniques, treatment planning systems, delivery techniques, and quality assurance protocols. In following studies, we will determine the center-specific overall treatment accuracy by performing measurements at each center using state-of-the-art dosimetry techniques. By comparing measured to planned dose for each RT center as well as dose distributions across all centers, we seek to gain a better understanding of the status of SIMT SRS and to provide guidelines for its application and QA to enable RT centers to use this technique in a safe and standardized way.

## Materials and methods

### Study overview

In all, 23 RT centers participate in the study: 20 in Germany, 2 in Austria, and 1 in Switzerland. Among these are 3 CyberKnife (CK; Accuray, Sunnyvale, CA, USA) centers, which we included to compare them to standard C‑arm linear accelerators and to provide a more complete picture of the current pattern of practice for SIMT SRS of multiple brain metastases. To recruit participating centers, the study was presented at WG STX DGMP meetings. Additionally, centers known within the working group’s network were contacted and invited to participate. The primary objective in selecting centers was to ensure comprehensive coverage of Germany, Austria, and Switzerland. A majority of 13 centers had prior clinical experience with SIMT SRS, while the remaining 10 centers are interested in its implementation and are using this study to benchmark their preliminary protocols. Therefore, the analyses presented distinguish between these two groups (experienced and benchmarking).

**Fig. 1:**
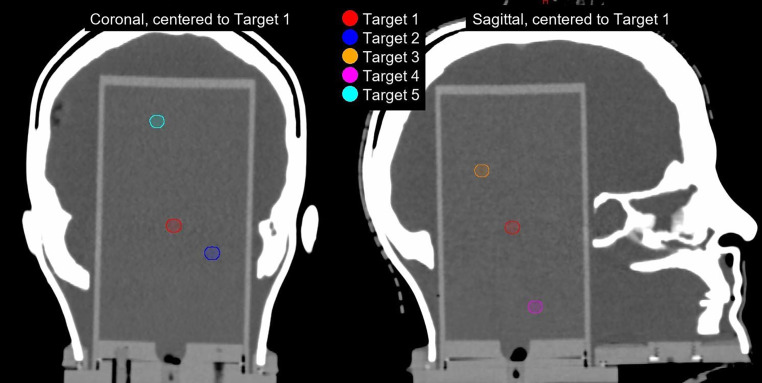
Computed tomographic (CT) representation of the anthropomorphic head phantom (RTsafe, Athens, Greece) with gel insert and reference structure case, including five target volumes. Target 1 is positioned near the center of mass of all targets, while other targets are progressively further away, with distances corresponding to their labels (e.g., target 2 is approximately 2 cm from target 1)

We defined a reference case based on a three-dimensionally printed anthropomorphic head phantom (RTsafe, Athens, Greece) designed to accommodate various detector inserts. For this pseudo-patient, we defined a reference structure set including five spherical target volumes (8 mm in diameter), representing brain metastases at varying distances from the presumed isocenter (Fig. 1). To achieve greater distances between the targets and due to limitations regarding our measurement volume, some targets had to be placed outside the hypothetical brain volume.

Throughout the course of this project, all centers were asked to provide their SIMT SRS protocols for this reference case, which are presented here in a pattern-of-practice analysis. The centers will also be visited individually to conduct on-site end-to-end measurements, the results of which will be correlated with the employed protocols. The measurements and their implications will be presented elsewhere.

### Pattern of practice

Based on the assessed SIMT SRS parameters collected at each center, we seek to provide an informational overview of existing approaches. To collect the relevant data, centers were provided with the reference data set (Fig. 1) for performing SIMT SRS treatment planning, along with a questionnaire requesting the parameters that define their protocols. The parameters are presented and discussed in the following sections (see also Appendix).

In the following sections, the use of these parameters and their discussion specifically refer to the reference case (Fig. 1).

Additionally, we evaluated parameters related to SRS experience, such as the average number of SRS and SIMT SRS cases per year, as well as QA protocols, including imaging and treatment isocenter consistency checks. In this context, we define SIMT SRS as the simultaneous, single-isocenter treatment of at least four separate targets.

We define consensus or agreement parameters as those for which at least 80% of centers concur on their general use. Infrastructure and protocols were assessed for compliance with DGMP/DEGRO consensus guidelines for stereotactic RT [3, 4].

Where appropriate, we use Welch’s t‑test for normality and equal variances [13] and the Mann–Whitney U test [14] for statistical analyses, defining statistical significance as a *p*-value of 0.05 or lower.

## Results

**Fig. 2:**
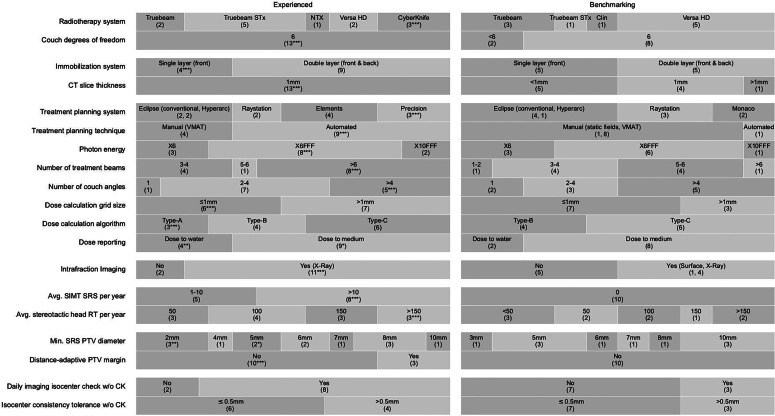
Single-isocenter multitarget stereotactic radiosurgery (SIMT SRS) infrastructure and protocol parameters across 23 radiotherapy (RT) centers (13 experienced and 10 benchmarking). The absolute number of centers using each technique is indicated in parentheses. Asterisks denote the number of included CyberKnife (CK) centers. Vendor names, system names, and abbreviations are detailed in the text

Collected parameters and data for the 23 RT centers are summarized in Fig. 2, whereby the following subsections refer to this figure.

### Radiotherapy system

There are 5 TrueBeams, 6 TrueBeams STx, 1 Clinac and 1 Novalis TX (Varian Medical Systems, Palo Alto, CA, USA), 7 Versa HD (Elekta, Stockholm, Sweden) and 3 CK systems. Apart from potential system-specific uncertainties, the type of treatment machine might be relevant for SIMT SRS application due to its specific multileaf collimator (MLC) system. The Varian TrueBeam STx and Novalis TX (NTX) have an MLC with a leaf width of 2.5 mm for the innermost leaf pairs and 5 mm for the outer leaves. All other C‑arm linear accelerators are equipped with MLC systems with 5 mm leaves throughout the entire field.

### Couch degrees of freedom

There is consensus in both groups to use a 6-degrees of freedom (DoF) patient couch. Since this parameter is primarily used to assess the capability for rotational alignment correction, and as the CK enables this through its robotic arm, the CK centers have been included in this group. Only two benchmarking centers use couch systems with 4 DoF.

### Immobilization system

There is consensus among the experienced C‑arm centers to use double-layer mask systems. Double-layer masks are mandatory when using Brainlab ExacTrac or Varian HyperArc, which are predominantly used by this group.

### CT slice thickness

There is consensus in both groups to use a slice thickness of 1 mm or lower for the primary planning CT. Only one benchmarking center uses 2 mm. Interestingly, all experienced centers use 1 mm exactly, while five benchmarking centers use a thickness below 1 mm.

### Treatment planning system and technique

There is a large variety of treatment planning systems (TPS): 9 centers use Eclipse by Varian (including 3 HyperArc), 5 Raystation (Raysearch, Stockholm, Sweden), 2 Monaco by Elekta, 4 Elements (Brainlab, Munich, Germany) and 3 Precision (for CK by Accuray). Systems with dedicated automated planning capability for SIMT SRS (HyperArc, Elements and Precision) are predominantly used by the experienced group.

### Photon energy

The majority of centers use flattening-filter-free (FFF) beams, with X6FFF being the most commonly used option among both experienced and benchmarking centers.

### Number of treatment beams

Except for CK with over 100 and HyperArc with fixed 4, the number of treatment beams across the other centers is rather diverse. On average, centers plan on using 1.7 beams per couch angle (not counting CK and not discriminating between half and full arcs). The number of beams per couch angle is higher for centers with fewer couch angles and lower for centers with more couch angles.

### Number of couch angles

There is consensus among all centers to use noncoplanar treatment with at least two different couch angles. For CK centers, noncoplanarity is achieved through the use of the robotic arm, which is why they are included in the group using more than 4 angles.

### Dose calculation and reporting

A total of 14 centers use a calculation grid size of 1 mm or lower, while 7 centers use 1.25 mm, 1 uses 1.5 mm, and 1 uses 2 mm.

Calculation algorithms are often categorized into type A, type B, and type C, depending mainly on their capability to account for lateral particle transport [15–18]. Due to the variety of employed algorithms and their different versions in our study group, we adhere to this terminology. The 3 CK centers use type A (PencilBeam), 8 centers use type B (PencilBeam convolution/superposition, e.g., AAA by Varian or PB X by Brainlab, and collapsed cone algorithms), and 12 use type C algorithms (advanced modelling, e.g., AcurosXB by Varian and full Monte Carlo algorithms).

Six centers calculate and report dose to water, 17 to medium.

For all these parameters, there is no significant difference between experienced and benchmarking centers.

### Intrafraction imaging

There is consensus in the experienced group to use intrafraction imaging, whereas no consensus exists in the benchmarking group. Overall, 11 centers use Exactrac by Brainlab with X‑ray setup verification either at fixed Gantry angles and/or after couch rotation. One center uses MV imaging (Gantry 0°) after each couch rotation. One center uses surface tracking with C‑Rad (CRad, Uppsala, Sweden). The 3 CK centers use X‑ray image guidance. Seven centers do not use intrafraction imaging but rely on initial setup imaging.

### Average number of SIMT SRS per year

This parameter is used to distinguish between experienced and benchmarking centers. Experienced centers have clinically used SIMT SRS prior to this study and treat at least one patient per year with SIMT SRS. Only five C‑arm centers treat more than 10 patients per year.

### Average number of stereotactic head treatments per year

The annual number of stereotactic head treatments per center ranges from 0 to over 200. The majority of centers (20) perform at least 50 per year, indicating that our study group is well experienced in this field. On average, SIMT-SRS-experienced centers also perform more stereotactic treatments overall.

### Minimum accepted PTV diameter for SRS

**Fig. 3:**
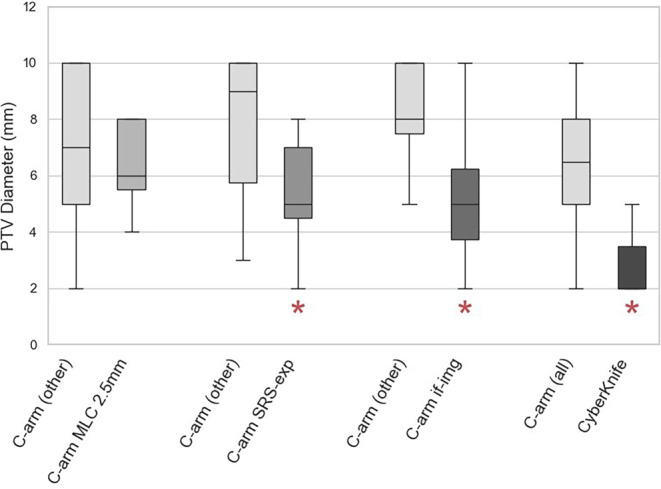
Comparison of minimum accepted planning target volume (PTV) diameters between subgroups: C‑arm MLC 2.5 mm referring to the employed multileaf collimator (MLC) system, SRS-exp indicating increased SRS experience (at least 2 per week) and if-img indicating the use of intrafraction imaging. C‑arm (other) is the reference group of all C‑arm centers, excluding the corresponding subgroups. Asterisks below the box indicate a significantly smaller diameter (*p* *<* 0.05)

Values range from 2–10 mm. Four subgroups were defined that could influence the average minimum accepted planning target volume (PTV) diameter *d*, and each was compared with the respective remaining group of C‑arm centers [13, 14]. CK centers (*d*_mean_ = 3 mm) accept significantly smaller diameters (*p* = 0.030) than the C‑arm centers (*d*_mean_ = 6.7 mm). Among C‑arm centers, there is no significant difference between 2.5 mm and 5 mm MLC systems. However, C‑arm centers with more SRS experience (≥ 2 SRS per week, *d*_mean_ = 5.3 mm) accept significantly smaller diameters (*p* = 0.043) than the remaining C‑arm centers (*d*_mean_ = 7.8 mm). C‑arm centers using intrafraction imaging (*d*_mean_ = 5.3 mm) accept significantly smaller diameters (*p* = 0.006) than those without it (*d*_mean_ = 8.3 mm). The comparison is illustrated in Fig. 3.

### Distance-adaptive PTV margin

A subgroup of 3 experienced centers uses distance-adaptive margins, where margins increase with increasing distance between target and isocenter. While 1 center only distinguishes between isocenter and off-isocenter (using 1 mm and 2 mm margin, respectively), the other 2 centers apply multiple incremental steps, resulting in a margin range from 0.5 mm and 1.0 mm at the isocenter, increasing to 1.5 mm and 2.0 mm for distances up to 7 cm.

### Daily imaging isocenter check

There is consensus in the experienced group to verify the consistency between treatment and imaging isocenter every day stereotactic treatment is scheduled (this refers to any stereotactic treatment, not necessarily SIMT SRS). Among the centers not checking this daily, the test frequency ranges from 2 days to 3 months.

## Discussion

There are numerous SIMT SRS approaches among the participating centers. Certain parameters, such as the linear accelerator model or the TPS, are predetermined and beyond the direct control of on-site staff. Others describe how these foundational systems are utilized and may depend on their specific configurations. Both categories of parameters provide valuable insights into individual SIMT SRS approaches within the broader framework. This discussion will examine these parameters in the context of the DGMP/DEGRO consensus on quality requirements for SRS [3, 4], where applicable.

Consensus and differences between experienced and benchmarking centers are highlighted in the results section (Fig. 2) and further discussed below. Although their protocols have not yet been clinically implemented, benchmarking centers were included in our pattern-of-practice analysis to reflect both established and emerging protocols.

For beam collimation and directions, either mechanical cones or an MLC with a maximum leaf width of 5 mm are recommended [3, 4]. The system should allow for noncoplanar treatment. All systems comply with these requirements and noncoplanar treatment is a consensus parameter in our study group. Only 3 centers (13%) do not make use of this functionality (1 experienced, 2 benchmarking). Consequently, a number of radiation fields of at least three is therefore also a consensus parameter. Based on our measurements and plan comparisons, we will assess the impact of coplanar treatment, especially on the clinical plan quality, and adjust the recommendations accordingly, if necessary.

For the CT slice thickness, the guidelines recommend 1 mm [3, 4]. This is also a consensus parameter in our study group and only 1 center uses a slice thickness of 2 mm (benchmarking). For patient positioning, an invasive head frame or thermoplastic masks are recommended [3, 4]. Daily in-room image-guidance and online correction capabilities are necessary for target localization and positioning. All systems in our study offer in-room imaging capabilities. However, some systems offer only pretreatment setup imaging and correction and are not suitable for intrafractional imaging.

For dose calculation, a grid size of 1–2 mm is recommended and used by all centers [3, 4]. Only 1 center uses 2 mm (benchmarking), all other centers use 1.5 mm or less. In this context, it is important to consider the grid size that was used for beam commissioning. For SRS, it is advisable to use the smallest commissioned grid size. Using grid sizes that have not been commissioned and verified, especially with regard to dose gradients and absolute dose, introduces additional uncertainties.

The diameter of the treatment unit isocenter sphere (or maximum variation of the isocenter during gantry rotation) should be ≤ 1 mm [3, 4]. All systems generally comply with this in their specifications.

The consistency of imaging and treatment isocenter is crucial for precise dose delivery and is recommended to be verified daily when any stereotactic treatment is scheduled [3, 4]. Among the experienced group, 77% of centers are aware of this recommendation and 85% comply with it (some without knowing), making daily isocenter consistency checks a consensus parameter among this group. In the benchmarking group, 70% are aware of the recommendations but only 30% comply with them. This likely stems from the additional time required for this test during the routine morning check. Although automated procedures like the Machine Performance Check on Varian systems can assist, these typically use tolerances above the precision required for SRS and do not address external imaging systems critical for intrafractional imaging in noncoplanar treatments. Reducing test frequency might be justified after accumulating sufficient stability data over time. However, external imaging systems relying on ceiling-mounted panels or cameras are vulnerable to mechanical disruptions, such as unnoticed collisions with patient beds. Such events could cause abrupt changes in system performance, underscoring the importance of frequent testing over reliance on long-term trends—not only for SIMT SRS but for stereotactic RT in general.

Intrafraction imaging is arguably the most clinically debated parameter. While recommended for use with thermoplastic masks [3, 4], its implementation varies within our study group. Overall, 70% of centers use some form of intrafraction imaging (94% of which with x‑rays, 6% with surface-guidance). Notably, this figure rises to 85% among experienced centers, where consensus exists.

Most centers generally adhere to the DGMP/DEGRO guidelines [3, 4]. However, there is significant variability in some individual parameters, especially isocenter consistency QA and intrafraction imaging. Possible effects on the treatment accuracy will be evaluated based on our measurements. Unfortunately, assessing the influence of intrafraction imaging will be limited to the effects of system uncertainties, since our phantom does not simulate patient movement. Another parameter showing notable variability is the minimum accepted PTV diameter, which is not specifically mentioned in the DGMP/DEGRO. CK centers accept significantly smaller PTVs than C‑arm centers. Among C‑arm centers, those with more SRS experience and those using intrafraction imaging tend to accept smaller PTVs. However, especially for C‑arm systems, we believe that the infrastructure alone should never be the basis for smaller target volumes, margins, or modification of other parameters. Instead, parameters should be chosen carefully based on a combination of the employed system and the commissioning and QA protocols that have been established for them. Centers performing more SRS and having invested in intrafraction imaging hardware are likely to have dedicated, rigorous QA protocols and greater confidence in their systems.

A subgroup of three experienced centers uses distance-adaptive PTV margins, anticipating potentially increased system uncertainties in off-center regions. Such approaches are frequently discussed within the stereotactic community, and no consensus was found in our study group. Although we did not specifically request this information from the centers, we assume that very few of them based their decisions on measurements with their individual system. Whether or not distance-adaptive margins are necessary will always depend on the individual on-site system. This effect and potential patterns for specific systems will be evaluated based on our end-to-end test measurements and incorporated into future guidelines.

During the course of the project, consistent feedback from participating centers highlighted a strong interest in understanding how other centers approach the implementation of SIMT SRS. All centers demonstrated openness and a critical attitude toward their own protocols, acknowledging the lack of clear guidelines or recommendations in this area. This feedback reinforced the core motivation behind our pattern-of-practice analysis: to provide a comprehensive overview of current approaches, to assist centers in benchmarking their protocols, and to contribute to the development of new, standardized practices. The necessary recommendations and guidelines will be developed in consensus by the involved DGMP and DEGRO working groups, based on the combined results of this study and subsequent studies.

## Summary and outlook

We evaluated single-isocenter multitarget stereotactic radiosurgery (SIMT SRS) infrastructure and protocols from 23 radiotherapy centers in Germany, Austria, and Switzerland and presented an overview of the current pattern of practice. There is a large variety of different approaches regarding the employed systems as well as the parameters and techniques they are being operated with. This highlights the absence of clear, dedicated guidelines for SIMT SRS. In future studies, individual protocols will be correlated with measurement results from end-to-end tests conducted at each specific center. This analysis will assess their impact on dose application accuracy and clinical plan quality, forming the basis for developing recommendations to ensure the safe implementation of SIMT SRS.

## Appendix

### Questionnaire for SIMT SRS protocols

This is the questionnaire used to collect SIMT SRS protocol parameters from participating centers.

Specific for our study:

- Site name
- Immobilization mask system (vendor and name)
- Computed tomography (CT) slice thickness (mm)
- Treatment planning system
- Employed auto-planning tool, if any
- Dose calculation algorithm
- Dose reporting (to water or to medium)
- Dose calculation grid size (mm)
- Planning target volume (PTV) margin employed in our study
- Dose prescription protocol (e.g., Gy to surrounding isodose level)
- Photon energy (including FF/FFF)
- Location of isocenter (e.g., center of mass of target x)
- List of couch angles employed in our study
- Number of beams employed in our study
- Radiotherapy system (linac vendor and name)
- Couch system (including number of degrees of freedom [DoF])
- Imaging system (on-board and/or external) employed in our study
- Imaging protocol (setup and/or intrafractional, including intrafractional imaging scheme)
- Dose output on the day of measurement (deviation from reference conditions?)

General QA and experience parameters:

- Approximate number of annual SIMT SRS cases
- Approximate number of annual head stereotactic treatments (STX) cases
- For how many years have you been performing stereotactic radiotherapy (RT) in the head region?
- Diameter of isocenter circle from the most current star shot for gantry, collimator and couch
- Do you perform a complete Winston-Lutz test (e.g., following DIN 6875‑2, section 7.3.1.2) on a regular basis? If so, what are your tolerances?
- How often do you verify the coincidence of imaging and irradiation isocenter and what are your tolerances?
- What is the most recent deviation (with regard to the day of measurement)?
- Are you aware of the DGMP/DEGRO consensus statement for system requirements for stereotactic RT (10.1007/s00066-020-01603-1)?
- What is your smallest clinically accepted PTV diameter (mm)?
- Would you have treated the 5 targets (reference case) with SIMT SRS in your clinic or would you have used multiple isocenters due to the distances between targets?
- What is your largest clinically accepted distance between target and isocenter, if any?
- Do you use adapted, distance-dependent PTV margins in your clinic (e.g., small margins for targets close to the isocenter and larger margins for targets further away)?

## References

[1] Le Rhun E et al (2021) EANO–ESMO Clinical Practice Guidelines for diagnosis, treatment and follow-up of patients with brain metastasis from solid tumours. Ann Oncol 32(11):1332–1347. 10.1016/j.annonc.2021.07.016 34364998 10.1016/j.annonc.2021.07.016

[2] In: Journal of Clinical Oncology 40.12 (2022), pp. 1392–1392. 10.1200/jco.22.00593.

[3] Schmitt D et al (2020) Technological quality requirements for stereotactic radiotherapy. Strahlenther Onkol 196(5):421–443. 10.1007/s00066020-01583-2 32211939 10.1007/s00066-020-01583-2 PMC7182540

[4] Guckenberger M et al (2020) Definition and quality requirements for stereotactic radiotherapy: consensus statement from the DEGRO/DGMP Working Group Stereotactic Radiotherapy and Radiosurgery. Strahlenther Onkol 196(5):417–420. 10.1007/s00066-020-01603-1 32211940 10.1007/s00066-020-01603-1 PMC7182610

[5] Niranjan A et al (2019) Guidelines for Multiple Brain Metastases Radiosurgery. In: Niranjan A et al (ed) Leksell Radiosurgery. S. Karger AG, pp 100–109 10.1159/000493055 10.1159/000493055 31096242

[6] Rusthoven CG et al (2020) Evaluation of first-line radiosurgery vs whole-brain radiotherapy for small cell lung cancer brain metastases: the FIRE-SCLC cohort study. JAMA Oncol 6(7):1028. 10.1001/jamaoncol.2020.1271 32496550 10.1001/jamaoncol.2020.1271 PMC7273318

[7] Chang EL et al (2009) Neurocognition in patients with brain metastases treated with radiosurgery or radiosurgery plus whole-brain irradiation: a randomised controlled trial. Lancet Oncol 10(11):1037–1044. 10.1016/s1470-2045(09)70263-3 19801201 10.1016/S1470-2045(09)70263-3

[8] Welzel G et al (2008) Memory Function Before and After Whole Brain Radiotherapy in Patients With and Without Brain Metastases. Int J Radiat Oncol 72(5):1311–1318. 10.1016/j.ijrobp.2008.03.009 10.1016/j.ijrobp.2008.03.009 18448270

[9] Chao ST et al (2017) Stereotactic radiosurgery in the management of limited (1–4) brain metasteses: systematic review and international stereotactic radiosurgery society practice guideline. Neurosurgery 83(3):345–353. 10.1093/neuros/nyx522 10.1093/neuros/nyx522 29126142

[10] Kraft J et al (2019) Stereotactic Radiosurgery for Multiple Brain Metastases. Curr Treat Options Neurol. 10.1007/s11940-019-0548-3 30758726 10.1007/s11940-019-0548-3

[11] Palmer JD et al (2019) Single-isocenter multitarget stereotactic radiosurgery is safe and effective in the treatment of multiple brain metastases. Adv Radiat Oncol 5(1):70–76. 10.1016/j.adro.2019.08.013 32051892 10.1016/j.adro.2019.08.013 PMC7004936

[12] Uto M, Torizuka D, Mizowaki T (2022) Single isocenter stereotactic irradiation for multiple brain metastases: current situation and prospects. Jpn J Radiol 40(10):987–994. 10.1007/s11604-022-01333-7 36057071 10.1007/s11604-022-01333-7 PMC9529683

[13] Welch BL (1947) The generalization of ‘student’s’ problem when several different population variances are involved. Biometrika 34(1–2):28–35. 10.1093/biomet/34.1-2.28 20287819 10.1093/biomet/34.1-2.28

[14] Mann HB, Whitney DR (1947) On a test of whether one of two random variables is Stochastically larger than the other. Ann Math Stat 18(1):50–60. 10.1214/aoms/1177730491

[15] Wilke L et al (2019) ICRU report 91 on prescribing, recording, and reporting of stereotactic treatments with small photon beams: Statement from the DEGRO/DGMP working group stereotactic radiotherapy and radiosurgery. Strahlenther Onkol 195(3):193–198. 10.1007/s00066-018-1416-x 30649567 10.1007/s00066-018-1416-x

[16] Knöös T (2009) Evaluation and classification of dose calculation algorithms. AAPM Annual Meeting, Anaheim, CA (Presentation)

[17] O’Connor J (1957) The variation of scattered X‑rays with density in an irradiated body 10.1088/0031-9155/1/4/305 13452841

[18] Bassi S, Tyner E (2020) 6X Acuros algorithm validation in the presence of inhomogeneities for VMAT treatment planning. Reports Pract Oncol Radiother 25(4):539–547. 10.1016/j.rpor.2020.03.018 10.1016/j.rpor.2020.03.018 PMC7256059 32494226

